# A high recombination rate in eusocial Hymenoptera: evidence from the common wasp *Vespula vulgaris*

**DOI:** 10.1186/1471-2156-12-95

**Published:** 2011-11-04

**Authors:** Anu Sirviö, J Spencer Johnston, Tom Wenseleers, Pekka Pamilo

**Affiliations:** 1 Department of Biology, P.O.Box 3000, 90014 University of Oulu, Finland; 2 Department of Entomology, Texas A&M University, TAMU 2475, College Station, Texas 77843-2475, USA; 3 Laboratory of Entomology, Zoological Institute, Naamsestraat 59, B-3000 Leuven, Belgium; 4 Department of Biosciences, P.O. Box 65 FI-00014 University of Helsinki, Finland

## Abstract

**Background:**

High recombination rates have previously been detected in two groups of eusocial insects; honeybees and ants. In this study we estimate recombination rate in a eusocial wasp *Vespula vulgaris *that represents a third phylogenetic lineage within eusocial hymenopterans.

**Results:**

A genetic linkage map of *V. vulgaris *based on 210 markers shows that the total map length is 2129 cM and the recombination rate is 9.7 cM/Mb (or 103 kb/cM). The present estimate in *V. vulgaris *is somewhat smaller than in the honeybee *Apis mellifera *and intermediate between the estimates from two ant species (*Acromyrmex echinatior*, *Pogonomyrmex rugosus*). Altogether, the estimates from these eusocial species are higher than in any other insect reported so far.

**Conlusions:**

The four species (*V. vulgaris*, *A. mellifera, A. echinatior*, *P. rugosus*) are characterized by advanced eusociality with large colonies, clear queen-worker dimorphism and well developed task specialization. They also have colonies with a single, normally multiply inseminated (polyandrous) queen. Benefits of genotypic diversity within colonies (e.g. through improved task specialization or pathogen and parasite resistance) may have selected for both polyandry and high recombination rate in such advanced eusocial insects.

## Background

Genetic relatedness, the probability of sharing genes that are identical by descent (IBD), between interacting individuals is a central variable in the genetic models of social evolution [1]. The coefficient of relatedness can be calculated on the basis of a pedigree by following the rules of Mendelian transmission. However, the segregation of alleles among the gametes produced by a diploid individual is a stochastic process and therefore the relatedness at a specific locus is a variable with a predicted mean and variance [2]. The variance at a single locus depends on the segregation, and the variance among loci depends on the amount of recombination. Based on these principles, Sherman suggested that the evolution of insect sociality should benefit from a high recombination rate [3]. His argument was that recombination reduces the variance of relatedness and makes it more difficult for nepotistic recognition alleles to invade the population. This argument applies mainly to a case where the society consists of a simple family with one single-mated mother queen, and the variation of relatedness among the offspring depends only on the segregation of alleles from this single mother. Templeton [4] pointed out that recombination can also favor social evolution under a quantitative genetic model (through effects on the variance of inclusive fitness within broods and on the nonadditive genetic components in quantitative traits) without the restrictions imposed by single mating and hypothetical recognition alleles.

At the time when Sherman and Templeton presented their hypotheses, the most conceivable way to estimate variation in the recombination rate among species was to compare chromosome numbers. The comparison showed that eusocial species have, on average, significantly higher haploid chromosome numbers than their non-social relatives, both in the Hymenoptera and in the Isoptera [3]. Although the comparisons could be criticized for not correcting for phylogenetic non-independence, the differences were clear. Among ants, the chromosome numbers vary widely and there is no general trend of increasing chromosome numbers in different ant lineages [5]. However, Schmid-Hempel [6] detected a positive correlation between the chromosome number and colony size in ants, indicating that advanced social life might be associated with recombination.

Sherman [3] also stated that increasing the crossing-over rate could have the same effect as increasing the chromosome number and predicted that "eusocial species should have higher crossing-over rates than nonsocial species". Since then, estimates of the crossing-over rate have been made in a number of insects, including eusocial species. The results from two species of honeybees, *Apis mellifera *[7-9] and *A. florea *[10], and two species of ants, *Acromyrmex echinatior *and *Pogonomyrmex rugosus *[11,12], showed crossing-over rates that were higher than in any other animal looked at so far, thereby lending support to Sherman's prediction. Sherman [3] suggested that genetic diversity underlying caste and task specialization of workers could be important for the evolution of the recombination rate. Recent studies have emphasized this possibility [11,13,14] even though empirical evidence is lacking. The hypothesis is based on the general effect of recombination in increasing the diversity of multilocus genotypes among the progeny. As the division of labor among eusocial insect workers can have a genetic component [e.g. 15], the hypothesis suggests that recombination helps to produce a larger number of multilocus combinations and the colony thus avoids a too narrowly specialized workforce.

Even though the available studies show a high recombination rate in the honeybees and ants, the comparison still suffers from the small number of data points which are not phylogenetically independent. Hence, the aim of the present study was to estimate the recombination rate in a representative of yet another group of highly eusocial insects, the vespid wasps, where eusociality has evolved independently from ants and bees. Our results demonstrate that the wasp *Vespula vulgaris *is also characterized by a high recombination rate, thereby lending further support for the hypothesis that this feature is likely shared by most advanced eusocial species.

## Results

**Figure 1:**
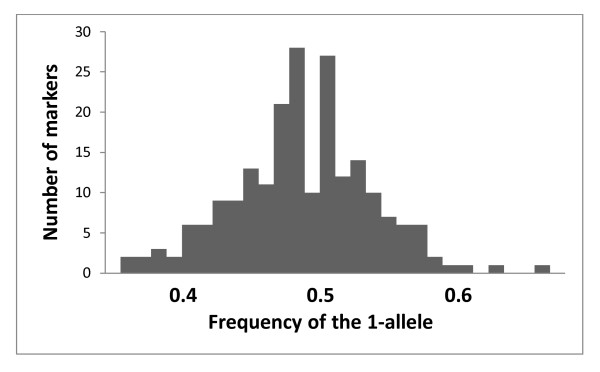
**The frequency distribution of the 1-alleles among the haploid male offspring**. The frequency is given as the proportion of males (total N = 86) carrying the 1-allele at the 210 AFLP marker loci.

We genotyped 86 males produced by the single wasp colony. The microsatellite genotypes (three loci) agreed with the assumption that the males were haploid sons of the colony queen. The 55 selective AFLP primer pairs revealed 217 polymorphic markers. At seven markers, we could not reliably score 11-13 individuals, at the other markers the number of unscored specimens was, on average, less than one. Length polymorphism was inferred from the presence of two mutually exclusive bands, and 32% of the markers showed length polymorphism. The mean frequency of the 1-allele over all the loci was 49.0%, and the frequency distribution (Figure 1) did not depart significantly from the symmetrical binomial distribution with the expected mean of 50% (Kolmogorov-Smirnov test, D = -0.076, P = 0.17).

**Figure 2:**
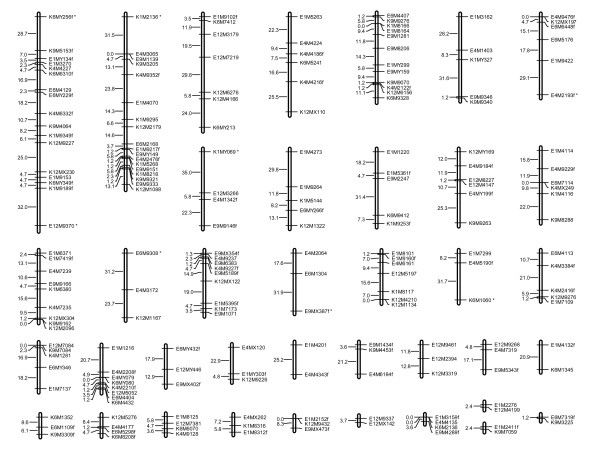
**Marker linkage groups of *Vespula vulgaris***. The map shows the linkage groups formed by 197 AFLP marker loci.

The informative AFLP markers were used to build linkage groups with Mapmaker [16]. Seven markers were discarded because they were tightly linked (0 cM) to the other markers derived with the same primer pair. Of the remaining 210 markers, 13 remained unlinked, and 197 were linked in groups with the final linkage thresholds of LOD score 3.0 and maximum marker distance 35 cM. The markers clustered in 38 linkage groups (Figure 2), six of which had only two markers. In total, eight originally unlinked markers could be integrated into the existing linkage groups with the near-command. Of the final linkage groups, two resulted from combining the preliminary groups (Figure 2).

With the error detection option of Mapmaker, our map (1760 cM) decreased by 4.9%; thus our map spans 1674 cM. The map was unsaturated because the haploid chromosome number of *Vespula vulgaris *(n = 25 [17]) was exceeded by 13 linkage groups. Since the number of linkage groups exceeds the haploid chromosome number, we added 35 cM (our threshold distance for joining the linkage groups) for each gap to cover the distance to the thirteen additional linkage groups. This resulted in a final recombinational map size of 2129 cM. However, it is notable that this underestimates the real distance and the recombination rate because the gaps in the linkage map may exceed the used threshold value of 35 cM.

The physical genome sizes estimated from the neural tissues were 219.8 ± 1.3 Mb for *V. vulgaris *(N = 4) and 219.6 ± 1.4 for the closely related *V. germanica *(N = 7). Males and females gave identical 1C estimates. As the total map length of *V. vulgaris *was estimated to be 2129 cM, 1 cM corresponds to approximately 103 kb, resulting in an estimated recombination rate of 9.7 cM/Mb.

## Discussion

Recombination by crossing-over is a genetic trait and the recombination frequency commonly varies both within and between populations [18]. As a result, the recombination rate can evolve and respond to natural selection. Laboratory experiments have demonstrated that the recombination rate can be selected readily, and directional selection has had a tendency to elevate recombination rates in domesticated animals and plants [18]. The findings that the recombination rate in the honeybees and ants is higher than in other insects [7-11] have led to suggestions that it has been selected for in eusocial insects. Our present results from *V. vulgaris *are in agreement with this suggestion even though the role of selection still remains to be demonstrated.

Beye et al. [9] estimated that the average recombination rate in the honeybee (*Apis mellifera*) genome is 19 cM/Mb with little variation among its 16 chromosomes (but with considerable variation of the local recombination rate along the genome). A somewhat lower estimate (16.0 cM/Mb) was calculated by Wilfert et al. [14]. Comparative genetic maps indicate that the recombination rate in another honeybee species, *A. florae*, is similar to that in *A. mellifera *[10], These estimates are four to five times higher than the estimate from the primitively eusocial bumblebee *Bombus terrestris*, 4.4 cM/Mb [19]. We earlier constructed linkage maps and estimated the recombination rates as 14.0 cM/Mb in the harvester ant *Pogonomyrmex rugosus *[12] and 6.2 cM/Mb in the leaf-cutting ant *Acromyrmex echinatior *[11]. The present estimate from the wasp *V. vulgaris *(9.7 cM/Mb) thus falls in between the two estimates from ants.

These estimates from the advanced eusocial bees, ants and wasps are higher than in other insects [14]. The estimates in non-social hymenopterans are within the range 2.5 - 5.4 cM/Mb (4 species of parasitoid wasps) and in other insects 0.1 - 6.1 cM/Mb (15 species) [14]. The recombination rates in the four advanced eusocial species (honey bee, leaf-cutter ant, seed harvester ant, yellow jacket wasp) are significantly larger than in the other hymenopterans (including the bumblebee, probability of no overlap in the values is P = 0.016) or in other insects in general (P = 0.0005). Bees, ants and wasps belong to aculeate Hymenoptera and are thus not phylogenetically independent. However, the lineages have diverged a long time ago and eusociality has evolved separately in them. According to Brady et al. [20], ants, bees and wasps had a common ancestor about 160 Mya, and the lineages leading to ants and eusocial wasps diverged about 140 Mya. The two ants in which the recombination rate has been estimated had their common ancestor about 80 Mya [21], and *Apis mellifera *and *A. florea *at least 8-10 Mya [10] and probably about 20 Mya [22]. Even though we cannot exclude the possibility that a high recombination rate is an ancestral state, there has been ample time for selection to modify the rates if they had any adaptive significance.

The use of AFLP-marker data deserves discussion because the methodology has gained criticism e.g. due to frequent occurrence of non-homologous fragments with the same amplicon length [23,24]. Study on *Nasonia *jewel wasp [25] revealed 41.5% shorter map size when SNP-markers were used instead of RAPD/AFLP markers [26]. Similarly in *Bombyx mor*i silk moth recombination map estimates vary from 3432 cM (simple sequence repeat; [27]) to 1413.4 cM (SNP; [28]) depending on the method and the number of the markers. On the other hand the original estimate of the honeybee map size was based on RAPD markers (3500 cM [7]) and the subsequent estimates based on microsatellites (4000 cM [29]) or genome sequencing (4553 cM [9]) have not decreased it. There is thus no universal trend that the RAPD/AFLP markers would overestimate recombination, and the data from most insects used in our comparisons were obtained with these methods, making the results comparable.

Sherman [3] suggested that a high recombination rate could be adaptive in eusocial insects either because recombination equalizes the fractions of genomes shared by colony members or because it generates a larger number of different multilocus genotypes. Sherman particularly considered the advantage of genotypic diversity in caste and task specialization, and Schmid-Hempel [30] suggested that the same can also apply to defense against parasites. As noted by Schmid-Hempel, both recombination and multiple mating by females increase the genotypic diversity among the offspring and can be beneficial to eusocial insect colonies under selection by parasites. The difference between the two factors is that unlike recombination, mating with many males also increases allelic diversity. Multiple mating by queens is known in many eusocial insects but the average mating frequency is generally rather low [31]. It is noteworthy that the species in which high recombination rates have been estimated, have all monogynous societies, i.e. societies with a single queen. They also have large colonies with clear queen-worker dimorphism and elaborate division of tasks among workers, and the queens are typically highly polyandrous. The estimated number of effective matings is up to 17.6 in the honeybee *A. mellifera*, 1.9 in *Vespula vulgaris*, 4.7 in *Pogonomyrmex rugosus *and 5.3 in *Acromyrmex echinatior *[31]. These estimates are clearly higher than the mean estimates for eusocial insects in general. One could thus suggest that these species benefit from genotypic diversity within colonies and that this has selected both for polyandry and for a high recombination rate

Sherman [3] initially hypothesized an association between recombination rate and sociality because of effects on genomic multilocus relatedness. The point is that the expected relatedness among full sisters (r = 0.75) in a single locus is in fact a mean between complete identity (r = 1) when the sisters received an identical allele from the mother and 'half identity' (r = 0.5) when the sisters received different maternal alleles and share only the paternal allele. Lack of recombination could result in genetic cliques within which sisters are unusually highly related over many loci. If there is any kin discrimination within the societies and nepotistic behaviour based on this discrimination, such genetic cliques could lead to nepotistic conflicts and harm the function of the colony. Nepotistic behavior based on kin discrimination has been doubted but some evidence for it has been presented recently [32,33]. Whether the effect of recombination on the distribution of pair-wise relatednesses among colony members could affect kin recognition and discrimination remains to be studied.

## Conclusions

The data from three major groups of eusocial Hymenoptera (ants, bees and wasps) show high recombination rates, supporting the theoretical predictions on the positive association between sociality and recombination. These predictions are derived from two separate lines of reasoning. First, recombination equalizes multilocus relatedness among brood members and prevents kin discrimination. However, kin discrimination is controversial and there is little evidence for it (e.g. [34]) and it is doubtful whether it could be a factor selecting for recombination. We therefore incline to the second hypothesis, according to which recombination is an important factor generating multilocus genotypic diversity within a society [3,30] and therefore highly relevant for the function of advanced eusocial colonies [11,13,14]. It can be hypothesized that benefits of intracolonial genotypic diversity has selected for both polyandry and high recombination rate. Hughes et al [35] found a negative correlation between polyandry and polygyny in data from 241 eusocial insect species. This suggests that polyandry and polygyny are alternative ways to increase the genetic diversity within societies. Consequently, we predict a negative correlation also between recombination rate and polygyny under the diversity hypothesis. Data from non-social species as well as from eusocial species with different colonial types (polygynous colonies, small colony sizes) are required for comparative tests of the adaptive role of recombination in advcanced eusocial insects.

## Methods

### Physical genome size

The size of the physical genome was determined from neural tissue of four *Vespula vulgaris *and seven *V. germanica *individuals (males and females), collected from nests that were dug up in Leuven in August 2009. Samples were prepared for flow cytometric genome size determination as described in [36]. Neural tissue dissected from the head of each sampled specimen was placed along with a single head of a female *Drosophila virilis *into 1 ml of cold Galbraith buffer in a 2 ml Kontes Dounce tissue grinder, stroked 15 times with the "A" pestle and then filtered through 20 μm nylon mesh. Propidium iodide was added to each sample to a final concentration of 50 ppm and allowed to stain 40 minutes in the dark on ice. To determine the relative positions of sample and standard fluorescence peaks and test for possible artifacts of preparation [37], similar preparations were made with at least one insect of each species alone. The mean fluorescence of stained nuclei in replicate samples was quantified using a Partec Cyflow cytometer with a 100 mw green laser tuned at 532 nm. Fluorescence at >615 nm was detected by a photomultiplier screened by a long pass filter. To ensure that scoring included only intact nuclei free from cytoplasmic tags, counting was activated by red fluorescence, and only nuclei with low forward and low side scatter were included in the analysis. Samples were run to produce a total of at least 1000 nuclei under each scored peak. DNA content was determined from co-preparations of sample and standard by multiplying the ratio of the mean peak fluorescence of the 2C sample to the 2C mean fluorescent peak of *D. virilis*, times the genome size of the standard (328 Mbp for *D. virilis*, based on co-preparations with *D. melanogaster*, n = 30).

### DNA extraction and quality, AFLP

**Figure 3:**
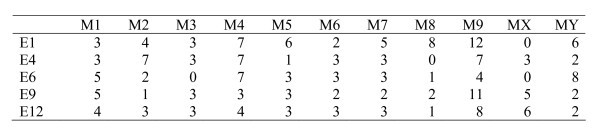
**The number of polymorphic markers produced by 55 different primer combinations (E × M)**. The total number of polymorphic markers used for mapping is 210.

Our mapping material consisted of *Vespula vulgaris *males collected as newly emerged adults from a single nest which had been dug up in Sint-Truiden Belgium, in September 2005. Samples were preserved in 99% ethanol. Along with these males, a queen and multiple workers were sampled for evaluation of the quality of male DNA, and to assure the haploid origin of the males. The quality of the DNA originating from different body parts (head, thorax, abdomen) of a few individuals was tested based on amplification using three microsatellite primer pairs (LIST 2001 F-R, LIST 2002 F-R and LIST 2003 F-R [38]). PCR-conditions were optimized for each microsatellite primer pair as follows: For LIST 2001 we used an annealing temperature of 52°C and 1.5 mM MgCl_2_, for LIST 2002 an annealing temperature of 47°C and 1.5 mM MgCl_2 _and for LIST 2003 an annealing temperature of 54°C and 1.0 mM MgCl_2_. The DNA from the thorax gave the best yield of PCR product. DNA was subsequently extracted from the thoraces of males, the mother queen and four workers with a Qiagen DNAeasy Tissue Kit. All the samples were tested with the microsatellite primers to confirm haploidy and maternity. Six males were discarded due to poor quality of DNA and one due to potential diploidy, and we were left with 86 haploid males. These males were further genotyped for mapping purposes using AFLP markers (see [39] for the method). These are commonly dominant (presence/absence) markers, which leads to a loss of information in a diploid population, but are fully informative in our study because we used haploid males. Samples were prepared by using the AFLP Core Kit for small plant genomes (Applied Biosystems) according to the manufacturer's protocol and were run on an ABI 3730 sequencer. Ten males were used for prescreening primer pairs that produce polymorphic markers. In the end 55 different combinations of EcoR1 and Mse1 primer pairs were used for the rest of the males (see Figure 3). AFLP data were analyzed using GeneMapper version 3.7 and the validity of all peaks was evaluated by manual inspection. Segregating markers were scored as 1 (allele present) or 0 (allele absent). Fragment length polymorphisms were scored by using the same system and marking the alleles as 1 ( long allele) or 0 (short allele). Any AFLP bands that were not clear were either rerun or marked as missing.

### Linkage analysis

Variable AFLP markers in haploid males were used to build linkage groups for *V. vulgaris *by using Mapmaker version 3.0 [39]. This was done using the phase unknown procedure as described in [11,40]. The Kosambi mapping function was used to transform the recombination fractions into centimorgans. This procedure reduces the map length by taking into account possible double recombination events. In two-point analysis, the preliminary linkage groups were formed with the thresholds of LOD score 5.0 and maximum marker distance 30 cM, and the order of markers within linkage groups was confirmed in multipoint analysis with the same criteria. When several other markers were already clustered and ordered in linkage groups, unlinked markers were added in the end parts of the groups with the criteria LOD 3.0 and distance 35 cM by using "near"-command that is based on two-point analyses [11]. Finally, some of the existing groups were combined if the distal markers were within 35 cM distance and the statistical threshold LOD score was at least 3.0. The order within these newly formed groups was tested again and the final marker linkage groups were formed with the criteria LOD 3.0 and 35 cM. The final map was then run through a genotyping error detection process of Mapmaker 3.0, with the error detection option switched on with an *a priori *error probability of 1% to detect the presence of multiple crossing-overs in linkage groups. As a final check, the raw data were inspected manually, and arranged according to the order of markers to allow visual identification of possible falsely scored individuals and by rechecking the interpretation of marker peaks for the loci and individuals.

## Authors' contributions

AS guided the laboratory work for AFLP, microsatellites, constructed the linkage map and drafted the manuscript. JSJ defined the physical genome size and modified the manuscript. TW collected the samples and modified the manuscript. PP supervised the study, did the statistical analyses and helped to draft the manuscript. All authors read and approved the final manuscript.
